# Evaluating the effectiveness of a 6-week hybrid mindfulness-based intervention in reducing the stress among caregivers of patients with dementia during COVID-19 pandemic: protocol of a randomized controlled trial

**DOI:** 10.1186/s40359-022-00876-8

**Published:** 2022-07-19

**Authors:** Patrick Pui Kin Kor, Meng Li Li, Denis Ka Shaw Kwok, Angela Yee Man Leung, Danial Lok Lam Lai, Justina Yat Wah Liu

**Affiliations:** 1 School of Nursing, The Hong Kong Polytechnic University, Hong Kong, China; 2 WHO Collaborating Centre for Community Health Services, The Hong Kong Polytechnic University, Hong Kong, China; 3 Hospital Authority, Hong Kong, China

**Keywords:** Mindfulness-based, Caregiver, Dementia, Online, Face-to-face

## Abstract

**Background:**

Mindfulness-based intervention (MBI), an emotion-focused approach, has been shown promising and sustainable effects on enhancing the well-being of caregivers of patients with dementia (PWD). However, the conventional MBI was quite demanding, had high rates of attrition and inconsistent long-term effect. The social distancing measures introduced during the COVID-19 pandemic also restricted face-to-face psychosocial intervention. The study aims to evaluate the effectiveness of a 6-week hybrid MBI in caregivers of PWD over a 6-month follow up.

**Methods:**

This is a single-blinded, parallel-group randomized controlled trial (RCT). Eligible participants from three local nongovernmental organizations (NGOs) will be randomly divided into intervention groups and control groups in a ratio of 1:1. The participants in the intervention group will receive 6 weekly 90-min group-based sessions delivered through a face-to-face and online approach. The participants in the control group will receive brief education on dementia care with the same group size, duration, and frequency as the sessions in the intervention group. Immediately after the intervention and at the 6-month follow-up, caring stress and other outcomes will be assessed. Besides, a focus group interview will be conducted to identify the strengths, limitations, and therapeutic components of the intervention from their perspectives. For quantitative data, intention-to-treat analysis and Generalized Estimating Equations (GEE) will be used. For qualitative data, content analysis will be used.

**Discussion:**

This proposed hybrid model of MBI has several advantages, such as lower duration, longer follow-up period and easier access by family caregivers. Also, physiological indicators (e.g., heart rate viability and neuropsychiatric symptoms) will be measured in this study to show the body change after MBI. The quantitative and qualitative data of this research can also benefit the development of online or hybrid MBI for caregivers of PWD during the COVID-19 pandemic. Despite these strengths, it does have practical challenges and limitations. However, this proposed intervention has the potential to benefit not only the participants, but also the researcher as well as public health providers.

*Trial registration*: NCT05242614. Registered on 2022-02-16, https://clinicaltrials.gov/ct2/show/NCT05242614

## Background

Dementia is a worldwide public health priority that currently affects over 50 million people around the world, the number is expected to increase to 152 million by 2050 [1]. Similarly, the number of dementia cases in Hong Kong is predicted to triple from 100,000 cases in 2009 to 300,000 in 2039 [2]. Dementia is a syndrome that features a progressive and irreversible decline in cognitive functioning. It impairs one’s independent functioning and affects one’s daily living [3]. Over 90% of patients with dementia (PWD) experienced a range of behavioural and psychological symptoms of dementia (BPSD) including wandering, depression, and agitation [4]. Family caregivers of PWD involve in multiple caregiving tasks, such as aiding everyday activities of PWD and managing their BPSD. They also need to balance these caregiving tasks with other demands, such as their own career and social events [5, 6]. The caregiving burden, uncertainty about the progression of the disease, and family conflicts contributed to a high level of caregiving stress in caregivers [7]. Such stress can last for a decade, or until the care recipients passes away. The prolonged and high levels of stress could also put caregivers’ physical health at risk, resulting in various problems such as insomnia, high blood pressure, poorer immune function, a higher risk of developing other psychological symptoms and cardiovascular events [8–10]. Consequently, the high level of stress and poor psychological health of the caregivers will further affect the dyadic relationship, which may worsen the BPSD of the PWD [11]. Reducing the stress experienced by family caregivers can improve their physical and psychological well-being, dyadic relationship, and build their resiliency to take care of their PWD for a longer period of time. Moreover, the social distancing restriction during COVID-19 led to the suspension and reduction of community-based, as well as home care services for PWD. PWD felt frustrated and expressed anger to caregivers for staying at home [12]. The mental well-being of the caregivers was significantly affected due to isolation and limited respite. A research reported that the pandemic further increased the caregiver of PWD’s stress and anxiety level, particularly for those caring for patients with severe dementia [13]. Thus, stress reduction for caregivers is not only recommended but an imperative, especially during the time of Covid-19 [14].

### Rationale for using mindfulness and the conceptual framework

**Fig. 1:**
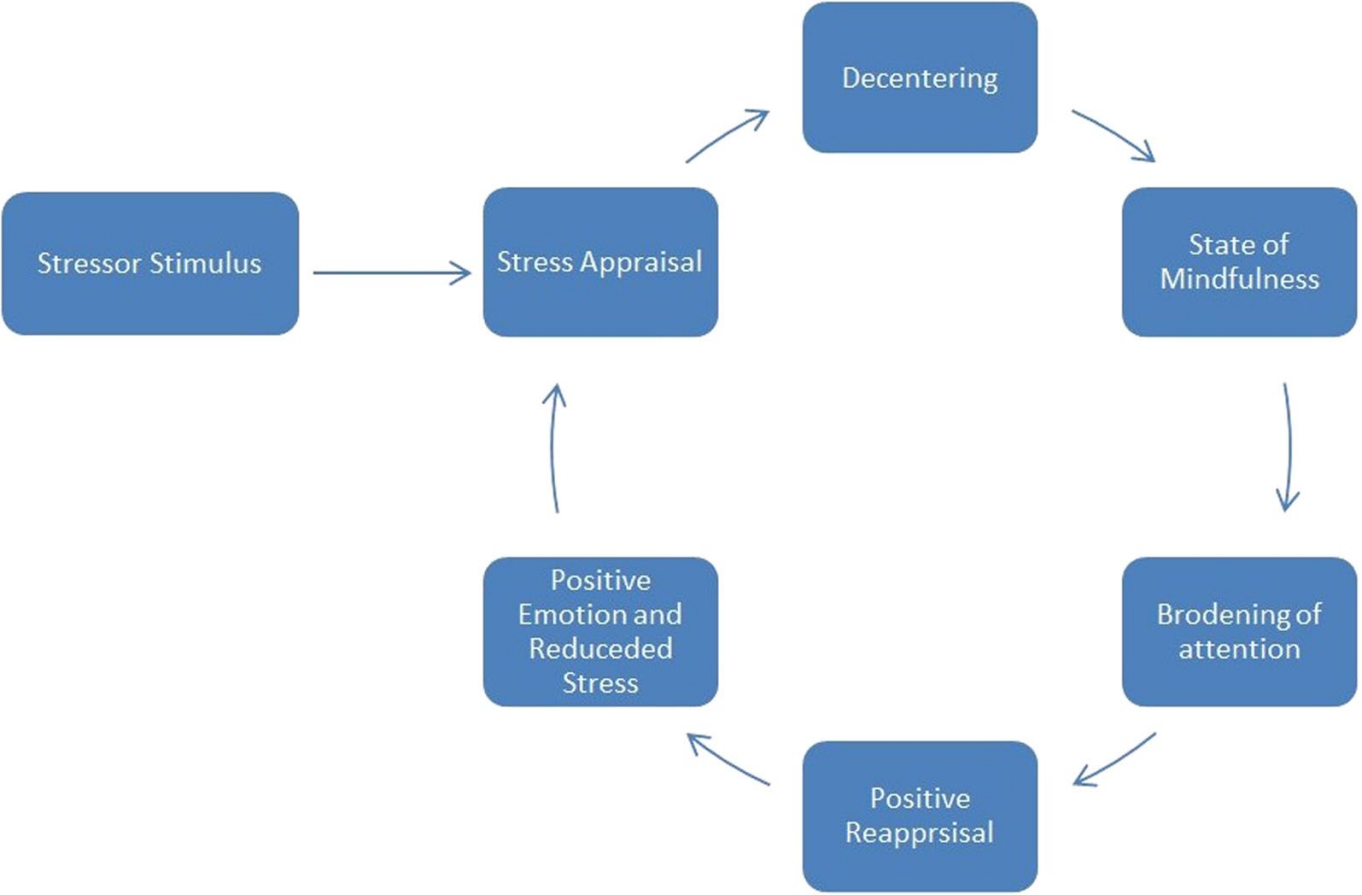
Mindful coping model (adopted from Garland et al. [18]; permission obtained from Elsevier License Number: 5301780254130)

In the recent decade, there has been growing attention to mindfulness due to its effectiveness in stress reduction. Mindfulness features the practice of awareness, attention, and remembering [15]. It includes different types of practices such as sitting mediation, mindful eating, body scanning and mindful walking. It is effective in improving psychological symptoms, including anxiety, depression, psychotic symptoms, and stress in various populations [16]. A recent meta-analysis of 131 randomized controlled trials found that mindfulness training has promising and sustainable effects on enhancing the well-being of caregivers of PWD, compared with other psychosocial interventions, such as counselling and support groups [17]. The potential mechanism of mindfulness in stress reduction can be explained by the mindful coping model (Fig. 1) [18]. As illustrated in the figure, mindfulness training can assist participants to step outside their negative experience and de-centre their sources of stress, and hence allowing caregivers to broaden their attention. Such a shift of attentional focus thereby allowed caregivers to generate a new meaning of the caregiving situations in the process of stress reduction and positive appraisal [19]. By objectively observing from a distance by stepping outside of the mind, participants will be able to realize that their mental events are not unchangeable truths, but only a constructed reality of the self [16].

Moreover, mindfulness and mediation are also useful for improving mental health during the COVID-19 pandemic. A research revealed that mindfulness practitioners, compared to non-mindfulness practitioners, reported having a lower level of depression, anxiety and pandemic-related distress [20]. Therefore, mindfulness training can be beneficial for caregivers to reduce stress during the pandemic.

### Existing challenges of applying the original MBI on family caregivers of PWD

Despite the usefulness of mindfulness, the feasibility of using the original MBI program on family caregivers of PWD is in doubt due to the intensiveness of the program. The original MBI protocol requires participants to commit over 27 h of practice within 8-weeks time. The program's intensiveness could potentially discourage family caregivers from participating, as they spent on average 21–63 h a week in taking care of the PWD [7].

A systematic review on the MBI program for family caregivers of PWD identified five MBI trials in the field [21]. Of these studies, most adopted the original MBI intervention (150-min weekly session for 8 weeks, together with 45 min of mindfulness practice per week and a 7.5-h retreat). Although this review suggested that the original MBI program had an immediate moderate effect in stress reduction on family caregivers, a high attrition rate was found ranging from 10.5 to 17.2%. Other mindfulness studies targeting family caregivers of PWD suggested similar challenges. Family caregivers shared their difficulty in attending the sessions and practicing mindfulness at home due to their busy caregiving schedule. Some also reflected that the contents should be relevant to dementia caregiving so that they can apply mindfulness skills in their daily caregiving tasks (e.g., responding mindfully to the PWD) [22]. The social distancing measures introduced during COVID-19 also created challenges for conducting face to face MBI program. The original MBI program involved group activities that may increase the risk of infection. All these findings and the social restriction during COVID-19 seem to suggest that there is an need to modify the original MBI program to better suit the needs of family caregivers of PWD and minimize the chance of social contact.

### The rationale for using a simplified version of mindfulness programme

Developing a more simplified and less intensive mindfulness program could be a viable solution. A recent review of the duration of mindfulness training showed there was no association between the number of in-class hours spent on mindfulness training and the psychological outcomes of various participants. This implied that a MBI program with a longer duration would not yield a more significant effect on the psychological outcomes of the participants after they had learnt and mastered the skills of mindfulness [23], suggesting that a original MBI program with a total of 27 training hours may not be necessary. A three-arm study conducted by Demarzo et al. also found that those who participated in either an eight or four sessions MBI demonstrated significant improvements in different psychological outcomes compared to the control (usual care) group at the 2 and 6 month follow-ups, with similar effect size (0.42 vs 0.45 in depression, 0.15 vs 0.13 in resilience, 0.46 vs 0.57 in anxiety) at the 6-month follow-up [24].

There are existing studies which adopted a less intensive MBI, and these studies seem to suggest a more positive finding regarding its feasibility and effectiveness for family caregivers of PWD. A 7-session group-based MBI program for family caregivers of PWD (total 14 h trainings) was carried out and evaluated in a RCT design [25]. Compared to the educational control group, the MBI group significantly reduced stress, depressive symptoms, and BPSD-related caregivers’ distress immediately after the program and 6-month after. The completion rate was over 83% and the attrition rate was only at 11.1%. Most caregivers could practice mindfulness 180 min per week. From the interviews of the same study, caregivers suggested a less intensive approach and a more flexible delivery mode can potentially lead to more widespread use of mindfulness in dementia caregiving.

In addition, the original MBI programme requires the involvement of a qualified interventionist who has undergone rigorous training in mindfulness and completed all of the modules (1 to 4) in a course for teachers of mindfulness or a person who has obtained a master’s degree from the Oxford Mindfulness Centre, which usually takes 2–3 years. This may impede the popularization of practising mindfulness for family caregivers of PWD. We agree that it is essential for an interventionist to receive rigorous training in order to deliver the MBI for a clinical population with some severe psychological problems (e.g., patients with severe depression). However, we suggested that a simplified version of an MBI protocol with a smaller number of sessions is needed for non-clinical caregivers of PWD to promote mindfulness practice.

### The rationale for using a hybrid programme of face-to-face and online delivery

The adoption of a more flexible delivery mode of the MBI intervention could make the program even more tailored to the needs of family caregivers. In recent years, the use of online MBI has gained much attention in the field [26]. However, a meta-analysis suggested that compared to face-to-face MBIs, the purely digitalized MBIs only had a limited effect in reducing family caregiver’s depression and anxiety symptoms (with an effect size of less than 0.4) [19]. The lower efficacy of the purely digital MBI can potentially be explained by the absence of in-person guidance given by the mindfulness therapist at classes. In face-to-face MBI, the mindfulness teacher/facilitator not only teaches mindfulness skills but also helps caregivers to learn and practise mindfulness in person [19]. Receiving mindfulness training through a purely online learning approach may not provide sufficient guidance to caregivers, and hence, caregivers may not learn and master mindfulness skills well.

Using a hybrid approach to deliver the MBI is a possible solution to this problem [27]. A meta-analysis of online education revealed that learning was more effective in hybrid and blended programs than face-to-face or purely online programs [28]. A hybrid MBI program contains both face-to-face and online features which can complement the strengths and weaknesses of face-to-face MBI sessions and online sessions. An online platform containing different self-directed learning materials makes it easier for caregivers to receive the training without interruption. Conversely, face-to-face sessions can provide caregivers opportunities to develop peer support through sharing their experiences and difficulties in practicing mindfulness with peers and the mindfulness facilitator. Moreover, the hybrid MBI program also minimizes the chance of group gathering and the risk of COVID-19 infection. It also provides flexibility for caregivers to attend the program as they need additional time for caregiving PWD at home during the pandemic.

In view of the limitations of previous studies, we will advance the intervention protocol and further investigate the scope of using mindfulness in dementia caregiving in this study. To overcome the challenges of time constraints imposed by the caregiving tasks and social distancing measures during the COVID-19 pandemic, we will adopt a simplified mindfulness protocol which includes only three face-to-face sessions and self-directed learning materials through hybrid mode. Furthermore, we have integrated several new components about dementia caregiving in the protocol such as mindful communication with people with dementia and exercises focusing on unpleasant caregiving experiences to strengthen the application of mindfulness in caregiving tasks. Based on the qualitative findings from our previous studies [25], we will also investigate the intervention effects on various caregiving outcomes such as dyadic relationship and positive aspect of caregiving that were rarely investigated in previous mindfulness studies. To the best of our knowledge, this is the first study investigating the effects of a simplified version of mindfulness programme for the family caregivers of PWD via hybrid mode.

## Methods

### Objectives

The objectives of the protocol are: (1) to examine the effects of a 6-week, hybrid (face-to-face and online) mode of a MBDCP on perceived caregiving stress of caregivers of PWD immediately after 6-week intervention and over a 6-month follow-up period; and (2) to identify the strengths, weaknesses and therapeutic components of the program from the views of family caregivers.

### Design

**Fig. 2:**
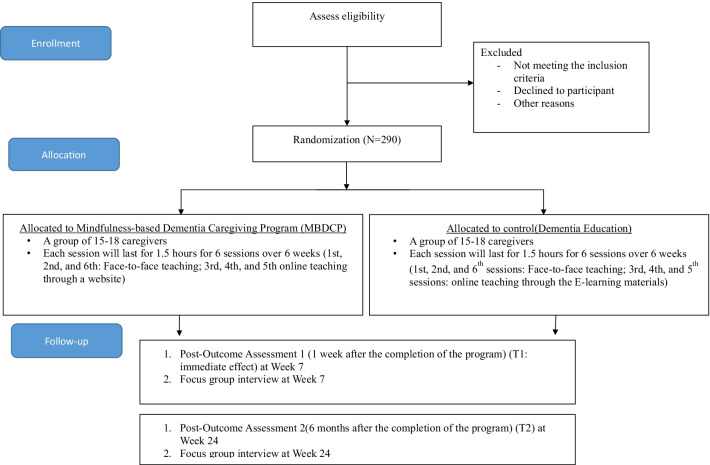
Consort Flow diagram of the study

**Fig. 3:**
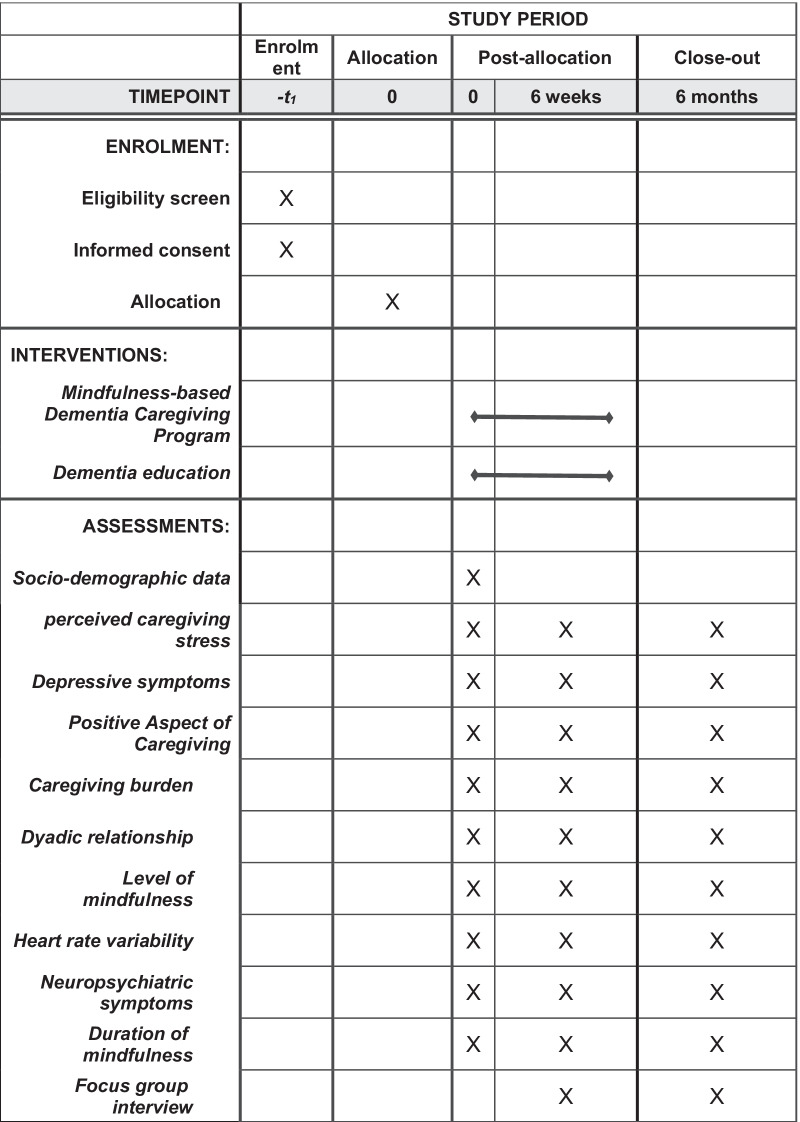
SPIRIT Flow diagram of the study

This protocol’s flow follows the CONSORT Diagram (Fig. 2) (http://www.consort-statement.org/)_and SPIRIT Flow Diagram (Fig. 3) (https://www.spirit-statement.org/).

This study adopts a mixed-method design to achieve the study objectives. A single-blinded, parallel-group randomized controlled trial (RCT) is used to evaluate the effect of the intervention (Fig. 2). Focus group interviews will be conducted to explore the participants’ experience attending the program and the short-term and long-term impacts on caregiving.

### Subjects and settings

In this study, family caregivers of PWD will be recruited from three local nongovernmental organizations (NGOs) that provide dementia care services (e.g., cognitive training, respite care) in Hong Kong. A family caregiver is defined as an unpaid individual who has a significant relationship with the PWD and is involved in providing assistance with daily life.

The participants will be eligible if they are family caregivers: (1) aged ≥ 18; (2) taking care of a family member who is diagnosed with dementia who has been residing in the community; and (c) providing care for at least 6 months prior to the subject recruitment. Caregivers will be excluded if (1) they have participated in any structured psychosocial intervention or mindfulness-based intervention/training in the 6 months prior to recruitment, (2) have acute psychiatric and medical comorbidities that are potentially life-threatening (e.g., suicidal ideation) or leave them with a limited ability to participate or adhere to the intervention (e.g., acute psychosis), or (3) do not have Internet access.

For the qualitative arm, purposive sampling will be adopted to select equal proportions of caregivers with different level of improvement in their stress (measured via Perceived Stress Scale) to join the focus groups.

### Sample size

The sample size is estimated based on the primary outcome of our study (perceived stress). This proposed study will adopt a simplified version of mindfulness training that differs from the protocol in our prior RCT [25]. Therefore, after considering the effect size of 0.36 in the prior meta-analysis on cognitive behavioural interventions for reducing stress in family caregivers of PWD [29], we adopted a conservative medium effect size of 0.40, which is similar to the effect size of psychological and behavioural interventions suggested by Cohen, to detect the mean difference in stress reduction between the intervention and control groups [30]. Considering the 20% attrition rate at 6 months that we found in our prior study, a sample size of 290 family caregivers (145 per group) is needed to achieve 80% power at 5% significance level (Two-sided). 20 caregivers from the intervention group will be purposively sampled to join in a focus group with a group size of 4 to 5 dyads per group one week after the intervention. After the intervention, equal proportions of participants with different levels (e.g., high, moderate, and low) of stress reduction will be selected.

### Interventions

The MBDCP was formulated by the principal investigator and his team (which includes a qualified MBCT therapist, clinical psychologist, nurses specialized in dementia care, social workers, and a psychiatrist) by modifying the prior MBCT protocol that was tested and adopted in our pilot and main study [22, 25]. The MBDCP includes 6 weekly 90-min group-based sessions delivered through a face-to-face (1st, 2nd, and 6th sessions) and online approach (3rd, 4th, and 5th sessions), as shown in Table 1. The online session is designed for self-directed learning. The caregivers will watch a teaching video (e.g., a video demonstrating mindfulness practices) through a website. The MBDCP includes different mindfulness practices (e.g. body scanning, mindful walking and mindful eating), psychoeducation on caregiving, and group sharing. The program aimed to enhance participants’ mindfulness skills through formal and informal mindfulness practice and help them to integrate these skills into their everyday life. Six sessions are adopted because in our prior studies showed that most caregivers were able to learn the mindfulness skills in the first four sessions, and two more sessions (5^th^ & 6^th^) were delivered to help the caregivers develop an action plan for further continuous practice. The duration of the intervention is shortened from 120 to 90 min because the in-class revision of practice will transform to online self-directed practice, in which all teaching materials and an audio recording of guided mindfulness activities will be provided to the participants through the website to enhance their daily practice and self-learning.

**Table 1:** Contents of the mindfulness-based Dementia Caregiving Program

Week	Teaching mode	Main theme	Contents (90 min per session)	Home practice
1	Face-to-Face	Caregiving stress and automatic pilot	Establishing the orientation of the class The raisin exercise (eating meditation) 13-min body scan Reaction to stress	45-min body scan for 6 out of 7 days Mindfulness of a routine practice
2	Face-to-Face	Thoughts and feelings	Exercises on thoughts and feelings related to dementia caregiving Mindful stretching and breath meditation Mindful movements 3-min breathing space	10 min of mindful breathing for 6 out of 7 days Pleasant experience calendar (one example daily) Mindfulness of a routine practice
3	Online	Facing difficulty with mindfulness	Exercises focusing on unpleasant caregiving experiences Practising seeing and hearing Sitting meditation (awareness of breath and body, and of responses to painful sensations)	40 min of mindful movements or stretching and breathing meditation for 6 out of 7 days Unpleasant experience calendar (a different experience for each day) 3-min breathing space, 3 times daily
4	Online	Mindful communication and responses	Practising seeing and hearing Mindful communication with people with dementia 3-min breathing space Responding to the behaviour of the care-recipient	Sitting meditation, 6 out of 7 days 3-min breathing space (3 times a day) 3-min breathing space – responsive (whenever one notices unpleasant feelings)
5	Online	Thoughts are not facts	Sitting meditation Exercises on thoughts and alternative viewpoints 3-min breathing space (responsive) Identifying habitual emotional reactions to difficulties resulting from caregiving	Select a guided meditation to practise at least 40 min per day 3-min breathing space (3 times a day) 3-min breathing space – added instructions (whenever one notices unpleasant feelings)
6	Face-to-Face	Taking care of yourself	Sitting meditation with breath, body, sounds Exploring difficulties in mindfulness Exercise on looking forward and preparing for the future	3-min breathing space (3 times a day) Select from all different forms of practice and apply them on a regular basis Develop actions to be used in the face of low moods

All of the contents in the Mindfulness-based Dementia Caregiving Program (MBDCP) will be closely related to dementia caregiving (e.g., instructions on how to respond mindfully to the behaviour of the care-recipient, exercises focusing on unpleasant experiences resulting from caregiving). Weekly online follow-up sessions provided by the facilitators via the website will be conducted to monitor the caregivers’ progress in learning and practising mindfulness. Drawing on the experience from our pilot study, the group size of the MBDCP group will be 15 to 18 participants to ensure adequate interactions between the mindfulness facilitator and all the caregivers. The caregivers can also communicate and share their practice experience with classmates via the online platform, as group discussion and mutual support is regarded as an active component of the MBI. All teaching materials, including the online video, will be prepared by a qualified MBI therapist. The qualified MBI therapist will mainly train the facilitators on how to deliver the three face-to-face training sessions. Drawing from a prior study on designing a Mindfulness Ambassador Programme [31], the facilitator will undergo a 21-h training (3 days) session to learn how to deliver mindfulness skills and how to answer the participants' questions. After the training session, the facilitator must pass a practical assessment by delivering two sessions of MBDCP to two groups of caregivers in the presence of a qualified MBI therapist, before being allowed to run the MBDCP. The criteria for the inclusion of the facilitator will be (1) aged 18 or above, (2) with at least 1 year of working experience in the field of social welfare, health care, or education (for better social and communication skills).

### Intervention fidelity control

All face-to-face sessions will be audio-recorded to check for fidelity to the intervention. After each session, an independent researcher with prior training in mindfulness will listen to the audio recording and monitor the intervention’s fidelity using a pre-designed fidelity checklist. This is to ensure that all the MBDCP sessions will be executed by the facilitator as intended. Based on the recommendations of the NIH Behavior Change Consortium [32], a fidelity rate of > 90% will be considered as acceptable. For online teaching, the website will guide the participants to watch each online video one by one. They will not be allowed to skip any sessions.

### Control group (Education on dementia care)

The family caregivers in the control group will receive brief education on dementia care which consists of 6 weekly 90-min group sessions, which will be delivered through a face-to-face (1st, 2nd, and 6th sessions) and online approach (3rd, 4th, and 5th sessions), with the same duration frequency and group size as the sessions in the MBDCP. The contents of the education offered to the control group will include brief education sessions on dementia care, caregiving skills, and group sharing. The group will be led by a registered nurse with experience in dementia care or elderly care.

### Instruments and measures

Data in this study will be collected via face-to-face data collection. The socio-demographic data will be collected at baseline (T0), while the outcome measurements will be collected at T0, immediately after the intervention (T1), and at the 6-month follow-up (T2). An independent assessor who is blinded to the group assignment (i.e. a research assistant with relevant experience and training) will carry out all these face-to-face assessments. Please find below the details of the outcome measurements:

### Primary outcomes measurement

#### Caregiving stress

Perceived caregiving stress will be assessed by the Chinese version of Perceived Stress Scale (PSS-10) [33]. It contains 10 items which measures the extent to which an individual has appraised their life as stressful. Respondents will be asked how often they felt in a certain way on a 5-point Likert scale from 0 to 4 (from never to very often). The total score ranged from 0 to 40, with a higher score indicating a higher perceived level of stress. The PSS-10 demonstrated satisfactory psychometric properties in the Chinese clinical samples [34].

### Secondary outcome measurements

#### Depression

Depressive symptoms will be assessed by the Chinese version of the Center for Epidemiologic Studies Depression Scale (CESD) [35]. It is a 10-item measure that asks respondents to rate how often they experience symptoms of depression (e.g. poor appetite, loneliness) over the past week. Respondents will be asked to indicate their response with a 5-point Likert scale from 1 (never) to 5 (very often). The total score ranges from 0 to 30, with a higher score representing more depressive symptoms. The CESD demonstrates satisfactory validity and reliability on the Chinese adults attending primary care services [36].

### Positive aspect of caregiving

Positive caregiving experience will be assessed by the Chinese version of the Positive Aspect of Caregiving Scale (PAC) [37]. The PAC is an 11-item instrument that measures the degree to which providing care to their relative with dementia has enabled them ‘feel important’ and ‘feel appreciated’. They will answer the questions on a 5-point Likert scale. The total score ranged from 11 to 55, with higher score indicating a more positive perception of caregiving. The PAC is a valid and reliable measurement among Hong Kong Chinese caregivers [38].

### Caregiving burden

Caregiving burdens will be assessed by the Chinese version of Zarit Burden Interview (ZBI) [39]. The scale comprises of 22 items which assess the subjective burden of caregivers. Respondents will be asked to rate their extent to which caregivers perceived their emotional or physical health, social life, and financial status to have changed as a result of caring for their PWD. The response option will be presented in a 5-point Likert scale (0 = None, 4 = Extremely distressing). The total score ranges from 0 to 88, with a higher score representing a higher level of caregiving burden. The Chinese version of ZBI is a valid and reliable instrument to evaluate stress experienced by caregivers of PWD in Hong Kong [40].

### Dyadic relationship

The Dyadic relationship is assessed by the Chinese version of Dyadic Relationship Scale -caregiver version (DRS) [41]; It is an 11-item scale that measures negative and positive dyadic interactions from the perspective of caregivers. The scale includes a four-option response (from 1 strongly disagree to 4 strongly agree). The scale generates two subscale scores: dyadic strain (ranges from 5 to 20) and positive dyadic relationship (ranges from 6 to 24). A higher score on each of these scales indicates a higher level of strain and positive interactions respectively. The DRS demonstrates satisfactory validity and reliability among caregivers.

### Neuropsychiatric symptoms of patients and corresponding distress

The neuropsychiatric symptoms of the care-recipients will be assessed by the Chinese version of Neuropsychiatric Inventory through caregivers [42, 43]. NPI-Q is an informant-based instrument that measures the presence and severity of 12 symptoms in patients with dementia and caregivers’ distress. The caregivers will be asked to identify whether the symptoms of the care recipients had been present in the past week and rate the severity of the symptoms (from a Likert scale ranged from 1 to 3) and the corresponding distress to them (from Likert scale ranged from 1 to 5). The NPI-Q provides two scores, namely, total severity of symptoms (ranges from 12 to 36) and total distress scores (ranges from 12 to 60). The higher scores indicate a higher level of symptoms severity of care recipients and distress of caregivers respectively. The Chinese version of NPI-Q is a valid and reliable measurement among stroke patients in Hong Kong [43].

### Heart rate variability

Heart rate variability (HRV) will be measured as a biomarker by using validated Polar heart-rate monitors (Polar Vantage M, Polar Electro Oy, Finland) [44]. HRV will be interpreted following the Guidelines for the Standard Measurement and Interpretation of HRV (European Society of Cardiology and the North American Society of Pacing and Electrophysiology) using the frequency-domain method [45].

### Level of mindfulness

Participants’ level of mindfulness will be assessed and served as a process indicator of this study as our program aims to reduce caregivers’ stress by enhancing their mindfulness level. The Chinese version of Five Facet Mindfulness Questionnaire-Short Form (FFMQ-SF) will be used [46]; FFMQ-SF is a 20-item questionnaire that measures the five facets of mindfulness: observing, describing and acting with awareness, nonjudging of and nonreactivity of inner experience. Items are scored in a 5-point Likert scale (from 1-Never to 5-Very often). The total score ranges from 20 to 100, with a higher score indicating a higher level of mindfulness. FFMQ-SF demonstrates good validity and reliability in both non-clinical and clinical population of Hong Kong Chinese [47].

Furthermore, participants will be asked to record the frequency and duration of their mindfulness practice once a week via the online platform. They will also be asked whether they had complied with the practice regime in the past 7 days, using a score ranging from 0 (Not complied at all) to 10 (Fully complied).

### Socio-demographic data

This section includes the followings: 1. age, gender, marital status, and level of education, living status; 2. health-related information, including medical history, activity of daily living (ADL) and instrument activity of daily life (IADL), cognitive status, and medications; 3. Use of social and caregiving support such as respite care, daycare centers, and domestic helpers.

### Qualitative arm

Two rounds of semi-structured interviews with a group size of 4 to 5 will be conducted immediately after the intervention and at the 6-month follow up. The focus group aims to explore the participants’ perception and experience in attending the program in terms of the strengths, weaknesses, therapeutic components of the mindfulness intervention, and also the short-term (e.g. immediately after the intervention) and longer-term (e.g. 6-month after the intervention) impacts on their caregiving. A guiding question will be employed, which will open up new testimonies: “What is your experience in joining the mindfulness program?” The participants will be then asked about how the experience impact their caregiving in different stages, the elements that they find that is useful, and the skills, knowledge, and any other benefits that they gain in the program during and after the intervention.

### Study procedure

Participants will be recruited via convenience sampling. Potential participants will be referred to the research team from three community center which provides services to the PWD (Collaborators of the research team). An independent Research Assistant (RA) will screen for their eligibility to participate in this study. A written informed consent will be sought from all the participants after the study was explained and all questions were answered. Following this, the participants will be interviewed about their health-socio-demographic information and complete the baseline assessment. Upon completion of the baseline assessment, they will be randomly allocated into the intervention group and control group (described in random allocation).

Follow-up assessments will be conducted by an independent RA who is blinded to the group assessing immediately after the 6-week intervention (T1), and after the 6-month post-intervention (T2). Focus group interviews will be conducted by within one week after T1 and at the 6-month follow up (T2) by an independent senior research assistant who has received training in how to conduct semi-structured interviews. Each focus group will last for about 60 min.

To ensure the RA can act as an independent outcome assessor throughout the stud, he/she will receive prior training on all the outcome instruments and interview techniques. The RA training will be provided by the PI. It consists of role play and on-site assessment practice with different clinical vignettes. Prior to the start of the study and throughout the data collection period, the scores rated by the assessor and the RA will be compared. The inter/intra-rater reliabilities will be evaluated by intra-class correlations (ICC). An ICC > 0.9 will be considered as an acceptable level of reliability.

### Random allocation and allocation concealment

Permuted block randomization will be employed in this study following the allocation concealment mechanism. Using an online, computerized sequence generation randomization tool (www.randomizer.org), an independent RA will generate a list of the permuted block sequences of two group labels (1 = MBDCP group, 2 = control group) in a 1:1 ratio. The participants will receive notice of their group allocation in an opaque sealed envelope on the first day of the intervention. The group allocation lists will be concealed from the researcher, the staff of the elderly centres, and the outcome assessors.

### Ethical considerations

Ethical approval will be obtained from the Ethics Review Committee of The University and study venues, respectively. The research team will comply with all of the requirements of a study involving human subjects as stated in the Helsinki Declaration and subsequent updates and the Good Clinical Practice guideline. No adverse effects from practicing mindfulness were reported in our prior study. A data monitoring committee will be formed and the group will consist of four independent experts from the fields of mental health nursing, psychology and gerontology. Throughout the study period, the committee will receive updates on participants’ outcome data (e.g., level of anxiety, stress, and depression). They will be contacted/ referred to health care professionals for further assessments if needed.

### Data analysis

SPSS version 23.0 will be used to analyze the data. An intention-to-treat analysis will be adopted. The demographic data will be presented in the format of descriptive statistics. Normality assumptions for the variables will be checked using the Kolmogorov–Smirnov (KS) test. Independent sample T-test (two-tailed) or Chi-square test will be used to examine demographic and clinical profiles between the two study groups. Generalized estimating equations (GEE) will be used to examine the study outcomes between the intervention and control groups across the three time-points (T0, T1, and T2). The dependent variables will be the mean total scores of the psychological health outcomes in the GEE analysis. The group, time points, and group x time interaction will be the independent variables. The missing data will be estimated in the GEE model based on the maximum likelihood estimation. Since there were no known covariates in our previous study, this assessment of homogeneity will be to identify any potential covariance in the outcomes. The possible covariates, such as age, gender, level of education, compliance, and psychiatric comorbidity, will be entered as covariates in a secondary analysis. All data analyses will be conducted with two-tailed tests with a significance level of p < 0.05.

For the interview data, content analysis will be performed using QSR NVivo 11. All audio recordings of the interviews will be transcribed into Chinese by the RA and the transcriptions will be checked for accuracy by the PI. To ensure trustworthiness, the transcripts will be coded independently by two Cantonese-speaking researchers. They will then discuss the codes by grouping them into main and subcategories with the support of verbatim data. The subcategories will then be re-organized. A set of subcategories with supporting verbatim data will finally be generated to identify the major strengths and limitations of the interventions as well as the long-term and short-term impact on caregiving. Any disagreements between the two researchers will be resolved through discussion with the larger research team.

## Discussion

Dementia is a prominent health issue globally and is expected to increase to 132 million by 2050 [2]. As the disease advances, the independent functioning of the PWD declines, and hence, the family caregivers of PWD have to take on various caregiving tasks. These caregivers also need to balance their caregiving duties with their other demands related to career and social life. Living in such a high stress situation caused not only poor psychological health, but also hampered the relationship between the caregivers and the PWD [7, 8]. Therefore, reducing the stress of family caregivers of PWD is crucial in dementia care.

Mindfulness has been shown promising and sustainable effects in enhancing the well-being of caregivers by raising the participants’ self-awareness at the present moment [16, 48]. However, the original MBI was quite demanding for family caregivers of PWD and had high attrition rate due to the high intensity of the program (long duration and frequent sessions).

Therefore, the 6-week hybrid MBI for caregivers of PWD (MDBCP) is proposed based on our previous studies [22, 25, 23, 24] which used mindfulness to reduce the stress of caregivers. The hybrid program has the following advantages. Firstly, the duration of the entire program is shorter than the original MBI (6 weeks vs 8 weeks). This not only reduces the cost of the intervention, it also reduces the time burden of family caregivers, whilst ensuring the effectiveness of MBI in stress reduction. Secondly, a hybrid model (combination of online and face-to-face), compared to the original MBI, is safer and more accessible to family caregivers as the busy schedule of caregivers and the risk of COVID-19 infection may prevent them in attending regular face-to-face sessions. The participants can still get sufficient supervision and peer support by using the hybrid model, particularly in the three face-to-face sessions. Thirdly, the physiological indicators (e.g., heart rate viability and neuropsychiatric symptoms) will be also measured in addition to stress, mindfulness level, dyadic relationship and so on. These parameters are essential to help us to better understand the body change after MBI and may shed light on the mechanism of MBI on stress reduction among caregivers of PWD. Moreover, the group interviews can give us some unique opinions and practical suggestions from the participants’ perspectives, which is helpful to improve MBI in the future.

The reduction of in-person support services during the outbreak of COVID-19 started a digital revolution for dementia care [49]. Digital technologies, such as video conferencing platform, has become widely adopted in psychosocial intervention for caregivers of PWD. The effectiveness of internet-based and web-based mindfulness intervention for caregivers of PWD is well documented [50]. This study can further provide evidence on the feasibility and effectiveness of a hybrid psychosocial intervention for caregivers of PWD during the COVID-19 pandemic. The result of the MDBCP will also be useful and crucial for future development on online and hybrid care support for caregivers of PWD.

Despite the above advantages, we foresee there are practical challenges when conducting this study. First, a large samples size of 290 participants is required. Subject recruitment could be particularly difficult under the COVID-19 pandemic. But we believe the online component of our program, compared to the original face to face MBI, could attract more family caregivers to take part in the study. To maximize the recruitment, the recruitment strategy of this study should focus on the unique strength of the program in which the hybrid component can give caregivers more flexibility to take part in the study according to their schedule. Second, it is common for participants to have a relatively low motivation for online learning compared to face-to-face sessions due to the absence of an actual interventionist on site. Participants may skip some online materials or forget to attend some sessions. To minimize the impact of this, we designed some follow-up sessions between each online session to prompt participants to complete the sessions. Third, in this study, only the researcher is blinded to the group allocation, and hence, there is a chance of bias as participants are aware of their group allocation. But we attempted to minimize such bias by measuring the level of mindfulness of participants so that we can examine whether the intervention effect we observed is contributed by the enhancement of mindfulness.

Based on the situation during the COVID-19 pandemic and previous studies on mindfulness programs targeting caregivers of PWD, we addressed the limitations of the original interventions by modifying the protocol and switching to a hybrid mode of delivery. To our knowledge, no studies had eve evaluated the effect of a hybrid mindfulness program for this population. Therefore, if this hybrid program was found effective, the existing program can potentially be used as a standard intervention to support family caregivers of PWD. The 6-week hybrid mindfulness-based intervention has the potential to benefit the caregivers of PWD by improving their well-being.

## Data Availability

Not applicable at this point.

## Abbreviations

ADL: Activity of daily living
BPSD: Behavioural and psychological symptoms of dementia
CESD: Center for Epidemiologic Studies Depression Scale
DRS: Dyadic Relationship Scale
FFMQ-SF: Five Facet Mindfulness Questionnaire-Short Form
GEE: Generalized estimating equations
HRV: Heart rate variability
IADL: Instrument activity of daily life
ICC: Intra-class correlations
KS: Kolmogorov–Smirnov
MBCT: Mindfulness-based cognitive therapy
MBDCP: Mindfulness-based Dementia Caregiving Program
MBI: Mindfulness-based intervention
MBSR: Mindfulness-based stress reduction
NGOs: Nongovernmental Organizations
PAC: Positive Aspect of Caregiving Scale
PWD: Patient with dementia
PSS: Perceived Stress Scale
RA: Research assistant
RCT: Randomized controlled trial
ZBI: Zarit Burden Interview
